# Acute onset painful acral granuloma annulare: Work-up and therapeutic strategies

**DOI:** 10.1016/j.jdcr.2022.09.036

**Published:** 2022-10-29

**Authors:** Tejas P. Joshi, Madeleine Duvic

**Affiliations:** a School of Medicine, Baylor College of Medicine, Houston, Texas; b Department of Dermatology, University of Texas, MD Anderson Cancer Center, Houston, Texas

**Keywords:** acral granuloma annulare, acute onset, granuloma annulare, opioid dependence, painful, AOPAGA, acute onset, painful, acral granuloma annulare, GA, granuloma annulare

*To the Editor:* We read with great interest the report by Ahmad et al,^1^ who describe a case of acute onset, painful, acral granuloma annulare (AOPAGA) and review the existing literature on this rare entity. As the authors note, AOPAGA is a unique manifestation of granuloma annulare (GA), which typically does not present with pain. Here, we wish to comment on the work-up and management strategies for patients with AOPAGA.

Ahmad et al^1^ note that their patient’s AOPAGA was so painful that it impaired her everyday activities. Thus, the authors recommend that patients with AOPAGA be treated with systemic therapy in order to mitigate this pain. We agree that systemic therapy should be pursued, especially if AOPAGA is refractory to topical corticosteroids. However, we wish to recommend that physicians avoid the use of opioid medication in treating patients with AOPAGA. Recently, we have shown that GA is significantly associated with opioid dependence, but not with alcohol, cannabis, or stimulant use.^2^ Given the increased prevalence of opioid use in patients with GA, we suggest that physicians exercise caution in the management of AOPAGA and prioritize non-opioid pharmacologic strategies.

First, treatment strategies aimed at resolving the GA should be employed. Indeed, methotrexate, a therapeutic option proposed by the authors, represents one such management strategy, with a recent case-series of 15 generalized GA patients demonstrating a 60% response rate to methotrexate.^3^ However, methotrexate also carries the risk of myelosuppression and may not be the optimal therapy in immunocompromised at-risk patients. Recently, targeted immunomodulators such as apremilast^4^ and janus kinase inhibitors (eg tofacitinib)^5^ have been reported to lead to remission of GA and may also be considered as systemic agents for AOPAGA.

Furthermore, we applaud Ahmad et al^1^ for conducting an extensive work-up for auto-immune, metabolic, and neoplastic processes. Indeed, GA has been noted to be significantly associated with diabetes,^5^ and it is possible that some cases of “AOPAGA” may simply be GA associated with underlying diabetic neuropathy, in which case gabapentin and tight glycemic control would represent the most appropriate therapeutic strategy. Work-up and management of other etiologies of neuropathy (alcohol, B12 deficiency, HIV, and iatrogenic) should also be pursued. Additionally, patients with overlapping osteoarthritis (ie Heberden’s nodes) may also benefit from both pharmacologic (eg non-steroidal anti-inflammatory drugs) and non-pharmacologic (eg hand exercises, warm compresses) interventions.

Altogether, we emphasize that systemic therapies for management of AOPAGA are crucial as this condition may significantly impair quality of life. However, use of opioid medications should be avoided and a thorough work-up for other underlying causes of pain should to be performed.

## Conflicts of interest

None disclosed.
